# Enhanced pre-synaptic glutamate release in deep-dorsal horn contributes to calcium channel alpha-2-delta-1 protein-mediated spinal sensitization and behavioral hypersensitivity

**DOI:** 10.1186/1744-8069-5-6

**Published:** 2009-02-12

**Authors:** David Nguyen, Ping Deng, Elizabeth A Matthews, Doo-Sik Kim, Guoping Feng, Anthony H Dickenson, Zao C Xu, Z David Luo

**Affiliations:** 1 Department of Anesthesiology & Perioperative Care, School of Medicine, University of California Irvine, Irvine, CA 92697, USA; 2 Department of Anatomy & Cell Biology, School of Medicine, Indiana University, Indianapolis, IN 46202, USA; 3 Department of Pharmacology, University College London, London, WC1E 6BT, UK; 4 Department of Neurobiology, Duke University Medical Center, Durham, NC 27710, USA; 5 Department of Pharmacology, School of Medicine, University of California Irvine, Irvine, CA 92697, USA

## Abstract

Nerve injury-induced expression of the spinal calcium channel alpha-2-delta-1 subunit (Ca_v_α_2_δ_1_) has been shown to mediate behavioral hypersensitivity through a yet identified mechanism. We examined if this neuroplasticity modulates behavioral hypersensitivity by regulating spinal glutamatergic neurotransmission in injury-free transgenic mice overexpressing the Ca_v_α_2_δ_1 _proteins in neuronal tissues. The transgenic mice exhibited hypersensitivity to mechanical stimulation (allodynia) similar to the spinal nerve ligation injury model. Intrathecally delivered antagonists for *N*-methyl-D-aspartate (NMDA) and α-amino-3-hydroxyl-5-methylisoxazole-4-propionic acid (AMPA)/kainate receptors, but not for the metabotropic glutamate receptors, caused a dose-dependent allodynia reversal in the transgenic mice without changing the behavioral sensitivity in wild-type mice. This suggests that elevated spinal Ca_v_α_2_δ_1 _mediates allodynia through a pathway involving activation of selective glutamate receptors. To determine if this is mediated by enhanced spinal neuronal excitability or pre-synaptic glutamate release in deep-dorsal horn, we examined wide-dynamic-range (WDR) neuron excitability with extracellular recording and glutamate-mediated excitatory postsynaptic currents with whole-cell patch recording in deep-dorsal horn of the Ca_v_α_2_δ_1 _transgenic mice. Our data indicated that overexpression of Ca_v_α_2_δ_1 _in neuronal tissues led to increased frequency, but not amplitude, of miniature excitatory post synaptic currents mediated mainly by AMPA/kainate receptors at physiological membrane potentials, and also by NMDA receptors upon depolarization, without changing the excitability of WDR neurons to high intensity stimulation. Together, these findings support a mechanism of Ca_v_α_2_δ_1_-mediated spinal sensitization in which elevated Ca_v_α_2_δ_1 _causes increased pre-synaptic glutamate release that leads to reduced excitation thresholds of post-synaptic dorsal horn neurons to innocuous stimuli. This spinal sensitization mechanism may mediate at least partially the neuropathic pain states derived from increased pre-synaptic Ca_v_α_2_δ_1 _expression.

## Background

Chronic pain derived from nerve injuries, or neuropathic pain, is a common clinical problem affecting over 50 million people in the United States [1]. Neuropathic pain may manifest as spontaneous pain, pain resulting from stimuli that are normally innocuous (allodynia), and increased pain responses to suprathreshold stimuli (hyperalgesia), all of which affect adversely the quality of patients' daily-life. Unfortunately, neuropathic pain tends to be long-term, and difficult to manage due to the poor efficacies and severe side effects associated with current conventional analgesic treatments. It appears that neuropathic pain with various etiologies can lead to similar behavioral endpoints that response differentially to pharmacological agents, suggesting that different mechanisms may underlie specific neuropathic pain states deriving from different pathological conditions. Thus, the development of target specific, safe and effective agents for neuropathic pain management relies on new insights into mechanisms of different neuropathic pain states.

Findings from previous studies have indicated that peripheral nerve injury induces a dramatic upregulation of the voltage-gated calcium channel (VGCC) α2δ_1 _subunit (Ca_v_α_2_δ_1_) in sensory neurons and spinal dorsal horn [2-6], which correlates with neuropathic pain development [2,3,7]. Knocking down of injury-induced Ca_v_α_2_δ_1 _upregulation in dorsal spinal cord with intrathecal antisense oligodeoxynucleotides results in allodynia reversal in spinal nerve injured rats [7]. In addition, transgenic mice overexpressing Ca_v_α_2_δ_1 _in neuronal tissue display tactile allodynia similar to nerve injury models that can be blocked by intrathecal gabapentin [8], a drug that binds to the Ca_v_α_2_δ_1 _proteins with high affinity [9] and has anti-neuropathic pain properties in patients and experimental animal models [2,3,10-16]. Furthermore, blocking axonal transport of injury-induced Ca_v_α_2_δ_1 _from DRG neurons to their pre-synaptic terminals in spinal dorsal horn diminishes tactile allodynia in nerve-injured animals [7]. Together, these findings suggest that elevated Ca_v_α_2_δ_1 _likely contributes to behavioral hypersensitivity through a pre-synaptic mechanism at the spinal cord level. This hypothesis, however, has not been tested directly.

Since elevated Ca_v_α_2_δ_1 _in the transgenic mice has been shown to cause dorsal horn neuron hyperexcitability, behavioral hypersensitivity [8]; enhanced glutamate neurotransmission is an important mediator in neuropathic pain processing [17,18]; and elevated Ca_v_α_2_δ_1 _may modulate various calcium channels that are known to regulate spinal pre-synaptic neurotransmission [19-25], we examined if elevated Ca_v_α_2_δ_1 _mediated deep-dorsal horn neuron excitability and behavioral hypersensitivity through activation of glutamate receptors, and if so, whether it was mediated by a pre-synaptic or post-synaptic mechanism. We tested these hypotheses in the injury-free Ca_v_α_2_δ_1_-overexpressing transgenic mouse model showing dorsal horn neuron hyperexcitability and behavioral hypersensitivity without the influence from other nerve injury factors [8].

## Results

### A similar tactile allodynia state is developed in spinal nerve injured mice and injury-free Ca_v_α_2_δ_1 _transgenic mice

**Figure 1:**
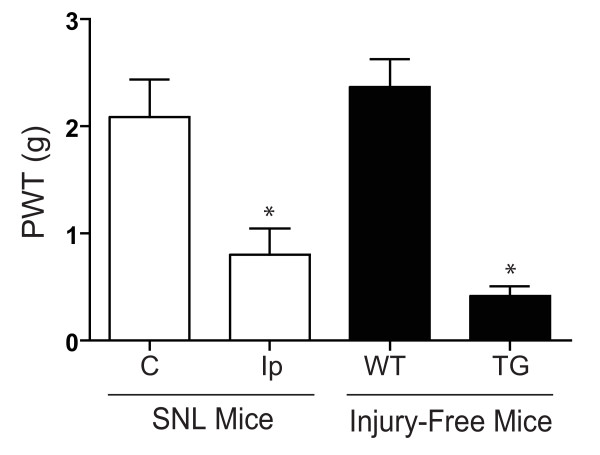
**Increased Ca_v_α_2_δ_1 _expression in spinal cord and DRG correlates with tactile allodynia in spinal nerve ligated and injury-free Ca_v_α_2_δ_1 _TG mice**. Unilateral L5 spinal nerve ligation (SNL) surgeries were performed in mice, and paw withdrawal thresholds (PWT) to von Frey filament stimulation were measured two-weeks post injury in SNL mice or in injury-free, adult male Ca_v_α_2_δ_1 _TG and WT mice as described in the Methods. Data presented are the means ± SEM from n = 6 for the SNL group, and n = 12 each for the injury-free WT and TG groups. C – contralateral (non-injury) side. Ip – ipsilateral (injury) side. * p < 0.05 compared to contralateral side or WT control using unpaired Students' *t *test.

Our previous studies have indicated that unilateral spinal nerve ligation (SNL) induces upregulation of the Ca_v_α_2_δ_1 _subunit in DRG and spinal dorsal horn that plays a critical role in tactile allodynia development [2,7]. However, nerve injury may also induce altered expression of other factors and activation of other pathways independent of the Ca_v_α_2_δ_1 _induction, which may also contribute to tactile allodynia development. To determine if injury-induced Ca_v_α_2_δ_1 _alone is sufficient to induce tactile allodynia, we examined the behavioral response of transgenic (TG) mice overexpressing the Ca_v_α_2_δ_1 _proteins in neuronal tissues [8]. Similar to the SNL mice (Fig. 1 left), the injury-free Ca_v_α_2_δ_1 _TG mice also developed a similar allodynic state compared with their wild-type (WT) littermates (Fig. 1, right). Since other factors in these two models, e.g. other injury-induced factors in the SNL model and compensatory factors in the Ca_v_α_2_δ_1 _TG mice, are not likely the same, these data support that elevated Ca_v_α_2_δ_1 _is highly likely a common factor mediating the abnormal sensations seen in these two models. This is supported by the findings that the tactile allodynia in both models can be blocked by treatment with intrathecal gabapentin [2,8,10], which binds to the Ca_v_α_2_δ_1 _subunit [9].

### A spinal glutamatergic pathway mediates behavioral hypersensitivity in the Ca_v_α_2_δ_1_-transgenic mice

**Figure 2:**
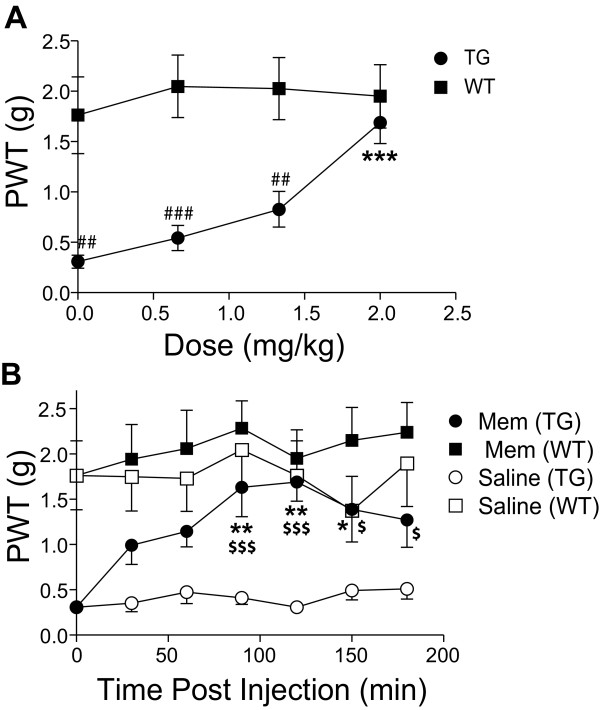
**Intrathecal NMDA receptor antagonists reverse tactile allodynia in the TG mice**. Memantine, an NMDA receptor antagonist, was injected intrathecally between L5/6 lumbar regions into adult TG and WT mice, and behavioral sensitivities to mechanical stimulation before or after drug treatments were assessed blindly as paw withdrawal thresholds (PWT) to von Frey filament stimulation in the hindpaw. **A**. Dose-dependent effects of intrathecal memantine for PWT in the TG and WT mice measured 120 min post i.t. injection. Data shown represent the means ± SEM from n = 7 for each group. ^## ^p < 0.01, ^### ^p < 0.001 compared with WT mice, *** p < 0.001 compared with the pretreatment level using two-way ANOVA followed by Bonferroni post-tests. **B**. Time-dependent effects of intrathecal memantine (2.0 mg/kg) on allodynia reversal in TG mice compared with saline treatment and similar treatments in WT mice. Data shown represent the means ± SEM from n = 7 for each group. * p < 0.05, ** p < 0.01 compared with the pretreatment level, ^$ ^p < 0.05, ^$$$ ^p < 0.001 compared with saline treatment in TG mice using two-way ANOVA followed by Bonferroni post-tests.

The pathway underlying Ca_v_α_2_δ_1_-mediated behavioral hypersensitivity is not known. Since activation of glutamate receptors plays a role in central sensitization in pain processing [18,26-29] and activation of selective glutamate receptors is believed to play a critical role in SNL-induced tactile allodynia [17,18], we tested the hypothesis that glutamate receptor activation might underlie the Ca_v_α_2_δ_1_-mediated behavioral hypersensitivity in the TG mice by examining the effects of intrathecal glutamate receptor antagonists on allodynia reversal in the TG mice. Intrathecal injection of memantine, a selective *N*-methyl-D-aspartate (NMDA) receptor antagonist, caused a dose-dependent allodynia reversal in the Ca_v_α_2_δ_1 _TG mice (Fig. 2A). Its anti-allodynic effects at the highest dose tested (2 mg/kg) lasted for two-three hrs (Fig. 2B), and diminished completely overnight (data not shown). Similar drug treatment in the WT littermates led to a slight increase of behavioral thresholds to the same type of mechanical stimulation, which was not statistically significant compared with the pretreatment level (Fig. 2). The allodynia reversal by memantine was similar to that reported in the SNL model [17].

**Figure 3:**
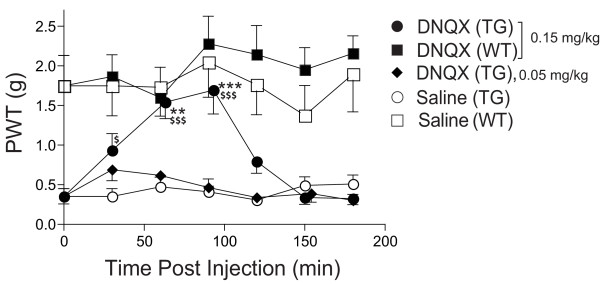
**Intrathecal AMPA/kainate receptor antagonists reverse tactile allodynia in the TG mice**. Different doses of DNQX, an antagonist of AMPA/kainate receptors, were injected intrathecally between L5/6 lumbar regions into adult TG and WT mice, and behavioral sensitivities to mechanical stimulation before or after drug treatments were assessed blindly as paw withdrawal thresholds (PWT) to von Frey filament stimulation in the hindpaw. Data shown are the dose- and time-dependent effects of intrathecal DNQX on allodynia reversal in TG mice compared with saline treatment or similar treatment in the WT mice. For the purpose of clarity, effects of the lower dose of DNQX (50 μg/kg) on PWT of WT mice, which were similar to that in saline treated WT mice, were not shown. Means ± SEM from n = 7 for each group. ** p < 0.01, *** p < 0.001 compared with the pretreatment level, ^$ ^p < 0.05, ^$$$ ^p < 0.001 compared with saline treatment in TG mice using two-way ANOVA followed by Bonferroni post-tests.

Intrathecal injection of 6,7-dinitroquinoxaline-2,3-dione (DNQX), a non-NMDA receptor antagonist for the α-amino-3-hydroxyl-5-methylisoxazole-4-propionic acid (AMPA)/kainate receptors, at a dose of 50 μg/kg provided little allodynia reversal in the TG mice. However, intrathecal DNQX at a higher dose (0.15 mg/kg) provided a rapid onset and transient allodynia reversal in the TG mice (Fig. 3), similar to that reported in the SNL model [17]. Similar DNQX treatment in the WT littermates led to a slight increase of behavioral thresholds to the same type of mechanical stimulation, which was not statistically significant compared with the pretreatment level (Fig. 3).

**Figure 4:**
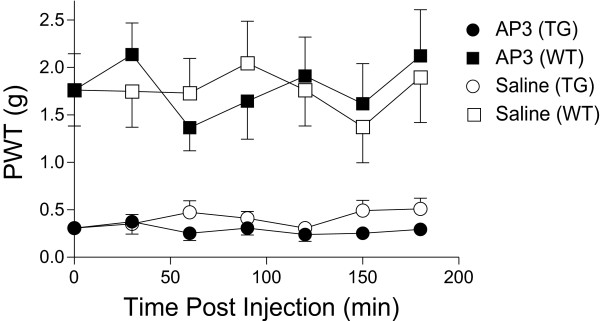
**Intrathecal antagonism of metabotropic glutamate receptors does not cause allodynia reversal in the TG mice**. AP3, a mGluR antagonist, was injected intrathecally between L5/6 lumbar regions into adult TG and WT mice, and behavioral sensitivities to mechanical stimulation before or after drug treatments were assessed blindly as paw withdrawal thresholds (PWT) to von Frey filament stimulation in the hindpaw. Data shown are time-dependent effects of intrathecal AP3, at the maximum tolerable dose (50 μg/kg), on PWT of TG and WT mice. Means ± SEM from n = 7 for each group.

Intrathecal injection of 2-amino-3-phosphonopropionic acid (AP3), a non-competitive metabotropic glutamate receptor antagonist, at a maximum tolerable dose reported (50 μg/kg) [17], did not significantly reverse tactile allodynia in the TG mice (Fig. 4), which was in agreement with findings from the SNL model [17].

### Ca_v_α_2_δ_1_-mediated spinal hyperexcitability through enhanced pre-synaptic glutamate release, but not likely through enhanced intrinsic excitability of dorsal horn neurons

We reported previously that elevated Ca_v_α_2_δ_1 _in the TG mice, without other injury induced factors, was sufficient to cause WDR neuron hyperexcitability and behavioral hypersensitivity [8]. Data from our previous studies have indicated that intrathecal injection of gabapentin, a drug that binds to Ca_v_α_2_δ_1 _proteins and has anti-neuropathic pain efficacy in the SNL model [2,3,10-12] and patients [13-15], can reverse the behavioral hypersensitivity in the Ca_v_α_2_δ_1 _TG mice [8], supporting that Ca_v_α_2_δ_1_-mediated WDR neuron activation contributes at least partially to the behavioral hypersensitivity. Since the wind-up response, a phenomenon of progressively exaggerated WDR neuron excitability to repetitive C-fiber stimulation, is similar between the TG and WT littermates [8], the hyperexcitability of the TG dorsal horn neurons is not likely mediated by a facilitated state mediated by the wind-up mechanism.

**Figure 5:**
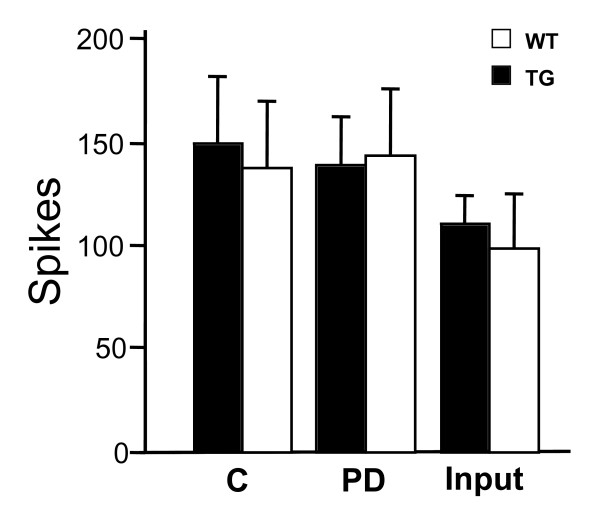
**Similar excitability to high intensity electrical stimulation between WT and TG wide-dynamic-range neurons**. Extracellular recordings were performed blindly to record dorsal horn single WDR neuron activity in response to C-fiber strength electrical stimulation applied to the receptive fields over the hindpaw of the WT and TG mice. Numbers of spikes from each neuron were pooled and the data are presented as the means ± SEM from n = 10 in each group. C – C-fiber responses. PD – post discharge.

To test if Ca_v_α_2_δ_1 _overexpression affects post-synaptic dorsal horn neuron excitability to acute stimuli, we compared C-fiber responses, post discharges, and non-potentiated responses (input) of WDR neurons from TG and WT littermates after brief transcutaneous electric stimulation with the C-fiber intensity. As shown in Fig. 5, similar levels of end point measures were observed between the TG and WT neurons. Even though our data do not allow the complete exclusion about possible influences from changes in strength of C-fiber synaptic inputs onto TG neurons, the occurrence of these changes, if any, would require opposite compensations in the excitability of TG neurons to maintain a level of excitability similar to that observed in WT mice, which are not supported by reported findings [8]. Thus, these findings suggest that the intrinsic excitability of WDR neurons, at least that to acute C-fiber inputs, is not changed in the Ca_v_α_2_δ_1 _TG mice.

**Figure 6:**
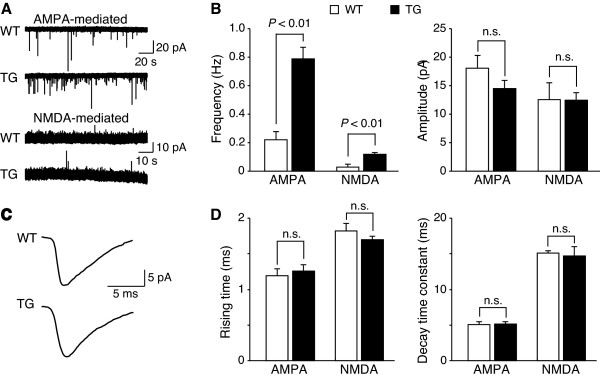
**Increased frequency, but not amplitude, of AMPA/kainate- and NMDA-receptor mediated mEPSCs in deep-dorsal horn neurons of the TG mice**. Spinal cord slices were prepared from the WT and TG mice, and spontaneous mEPSCs were recorded blindly from deep-dorsal horn neurons. **A**. Representative traces mediated by AMPA/kainate and NMDA receptors, respectively. **B**. Summarized mEPSC frequency (left) and amplitude (right) from WT and TG neurons mediated by AMPA/kainate and NMDA receptors, respectively. **C**. Representative traces of AMPA/kainite receptor mediated mEPSCs recorded from WT and TG neurons. **D**. Summarized mEPSC rising time (left) and decay time constant (right) from WT and TG neurons mediated by AMPA/kainate and NMDA receptors, respectively. Data presented are the means ± SEM from the number of independent neurons indicated in the text. Statistic significance is indicated by p < 0.01 using unpaired Students' t test. n.s. – not significant

This is further supported by our findings that elevated AMPA/kainate and NMDA receptor-mediated miniature excitatory postsynaptic currents (mEPSCs) in TG deep-dorsal horn neurons are at least partially mediated by enhanced pre-synaptic glutamate release, but not by changes in post-synaptic components, including enhanced intrinsic excitability of post-synaptic deep dorsal horn neurons. As shown in Fig. 6A and 6B, at a holding potential of -70 mV, the frequency of AMPA/kainate receptor mediated mEPSCs was significantly increased from 0.22 ± 0.06 Hz for WT neurons (n = 7) to 0.79 ± 0.08 Hz for the TG neurons (n = 9). Application of AMPA/kainate receptor antagonist CNQX (10 μM) could reduce mEPSCs by 91.3 ± 5.3% and 93.9 ± 3.3% in WT and TG neurons (n = 5 in each group, data not shown), respectively. In the presence of CNQX (10 μM), the frequency of NMDA receptor-mediated mEPSCs, recorded at a holding potential of +50 mV, was also increased in the TG neurons (WT: 0.03 ± 0.02 Hz, n = 5; TG: 0.12 ± 0.01, n = 3). However, the amplitudes of AMPA/kainate and NMDA receptor mediated mEPSCs were about the same between the WT and TG neurons. In addition, the kinetics of the AMPA/kainate and NMDA receptor mediated mEPSCs showed no obvious difference between the WT and TG neurons (Fig. 6C and 6D). The rising time of AMPA/kainate receptor mediated mEPSCs was 1.19 ± 0.10 ms (n = 7) and 1.26 ± 0.09 ms (n = 9) in WT and TG neurons, respectively, and the decay time constant was 5.05 ± 0.43 ms (n = 7) and 5.12 ± 0.39 ms (n = 9) in WT and TG neurons, respectively. The rising time of NMDA receptor mediated mEPSCs was 1.82 ± 0.11 ms (n = 3) in WT and 1.70 ± 0.05 ms (n = 3) in TG neurons, and the decay time constant was 15.13 ± 0.34 ms (n = 3) in WT and 14.70 ± 1.29 ms (n = 3) in TG neurons.

## Discussion

While previous evidences have indicated a clear correlation between nerve injury-induced Ca_v_α_2_δ_1 _upregulation and neuropathic pain states [3,4,7], pathways underlying Ca_v_α_2_δ_1_-mediated behavioral hypersensitivity have not been identified. Since cellular changes in spinal cord and DRG after a prolonged peripheral nerve injury include altered expression of large numbers of genes [5,6,30], multiple factors induced by nerve injury may contribute to neuropathic hypersensitivity. In an attempt to study if upregulated Ca_v_α_2_δ_1 _is sufficient to mediate behavioral hypersensitivity, and if so, what is the underlying mechanism, we have generated TG mice overexpressing Ca_v_α_2_δ_1 _in neuronal tissues and compared their behavioral sensitivity to mechanical stimulation with the SNL model. Our data indicated that the TG mice have tactile allodynia similar to that observed in the SNL model. Since similar behavioral hypersensitivity and increased Ca_v_α_2_δ_1 _expression occur in both models, and other factors such as compensatory changes in the TG mice and other injury-induced factors in the SNL model are not likely the same, our data, while do not allow the complete exclusion of other factors, strongly support that increased Ca_v_α_2_δ_1 _proteins are sufficient in mediating tactile allodynia observed in these models.

The pharmacology profiles in allodynia reversal by the spinally delivered, selective glutamate receptor antagonists are consistent with the pharmacology profiles of allodynia reversal by these agents in the SNL model as reported by others [17]. These data suggest that increased Ca_v_α_2_δ_1 _mediates allodynia through a pathway related to a selective activation of AMPA/kainate and NMDA receptors. In contrast, metabotropic glutamate receptor antagonist, AP3, was without effect in allodynia reversal. This is consistent with the notion that metabotropic glutamate receptors are mainly involved in acute and persistent pain states associated with input from afferent C-fibers, but less so on tactile allodynia likely mediated by input from Aβ fibers [31].

Since the TG mice have elevated Ca_v_α_2_δ_1 _in different neuronal tissues ranging from DRG in the peripheral to spinal cord and supraspinal cord locations in the central nervous system [7], it is possible that increased Ca_v_α_2_δ_1 _expression in peripheral as well as central locations in the TG mice contributes to the behavioral hypersensitivity through a glutamate receptor-dependent mechanism. Therefore, the enhanced dorsal horn neuronal excitability could derive from altered pre-synaptic neurotransmission, intrinsic post-synaptic neuron excitability or descending modulation from supra-spinal locations in the TG mice. While our data do not allow the exclusion of potential contributions to tactile allodynia by activated AMPA/kainate and NMDA receptors at supra-spinal locations in the TG mice, Chaplan et al. [17] reported that only spinal, but not i.c.v., injection of NMDA receptor antagonists reversed injury-induced allodynia in the SNL model. This suggests that activation of a spinal glutamatergic pathway is critical in the development of neuropathic pain states post peripheral nerve injury.

Data from this study further demonstrate that, in contrast to the enhanced excitability of TG WDR neurons to natural stimuli reported previously [8], acutely evoked excitability of WDR neurons to C-fiber-strength electric stimulation is not altered in the TG mice. While mechanisms underlying this discrepancy are not clear, the simplest explanation is that detected WDR neuron excitability to natural stimuli derives from neuronal activation by inputs from multiple sensory fibers including A-fibers, and elevated Ca_v_α_2_δ_1 _expression results in a higher excitability of the WDR neuron to A-fiber-, but not C-fiber-, strength stimulation (see discussion below). Thus, it is likely that increased Ca_v_α_2_δ_1 _expression mediates tactile allodynia through a Ca_v_α_2_δ_1_-dependent pathway by enhancing synaptic strength of A-fibers. Indeed, this hypothesis is consistent with reported findings that: (a) The basal level of Ca_v_α_2_δ_1 _expression is relatively high in small DRG neurons, low in large DRG neurons [6,8], and there is a dramatic increase in Ca_v_α_2_δ_1 _expression in large DRG neurons after SNL injury [6] or in the Ca_v_α_2_δ_1 _TG mice [8]; (b) increased Ca_v_α_2_δ_1 _expression correlates with development of allodynia mainly mediated by large Aβ fibers, but not formalin- and carrageenan-induced acute pain states mainly mediated by nociceptive C-fiber inputs in the TG mice [8], and formalin- or carrageenan-induced pain models do not have increased Ca_v_α_2_δ_1 _expression in DRG and spinal cord (data not shown); (c) increased Ca_v_α_2_δ_1 _expression in the TG mice does not alter the receptive field sizes of WDR neurons, nor the excitability of these neurons to repetitive C-fiber stimulation (wind-up) [8]; (d) In the model of sciatic nerve chronic constriction injury with increased Ca_v_α_2_δ_1 _expression [3], a subpopulation of dorsal horn neurons exhibits abnormal afterdischarges to mechanical and thermal stimuli, but the number of dorsal horn neurons showing wind-up phenomenon to repetitive C-fiber stimulation remains unchanged [32].

The increase in frequency of AMPA/kainate and NMDA receptor mediated mEPSCs in deep dorsal horn neurons from the TG mice suggests that elevated spinal Ca_v_α_2_δ_1 _proteins lead to an increased rate of quanta release of excitatory neurotransmitter glutamate from pre-synaptic terminals at the resting state, which in turn, enhances excitability of post-synaptic dorsal horn neurons through mainly AMPA/kainate receptor activation. Since the deep dorsal horn neurons for in vitro and vivo recordings were selected from a similar dorsal horn lamina, and very few non-WDR neurons (which were excluded) were detected in this lamina during neuron sampling in vivo, it is highly likely that a similar population of WDR neurons was examined in our in vivo and in vitro recording. Our data indicate that elevated Ca_v_α_2_δ_1 _proteins do not change the amplitudes of AMPA/kainate and NMDA receptor-mediated mEPSCs. This suggests that neither the quantum size of glutamate release nor the intrinsic excitability of post-synaptic dorsal horn neurons to glutamate receptor activation is changed by increased Ca_v_α_2_δ_1 _expression. Direct measurements of glutamate release in deep dorsal horn and membrane excitability of WDR neurons from TG mice would further help to clarify these issues.

**Figure 7:**
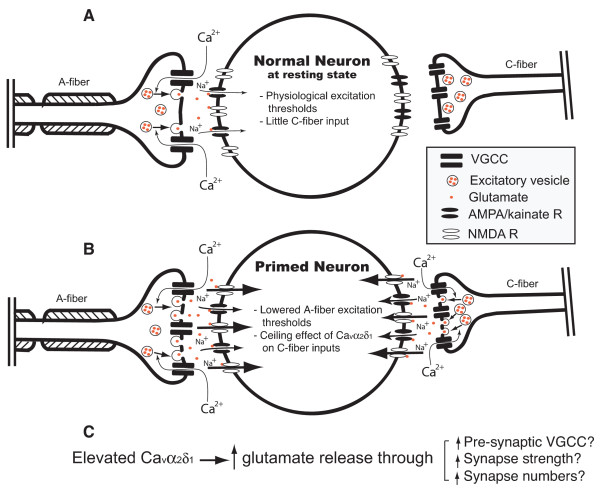
**Proposed mechanism of Ca_v_α_2_δ_1_-mediated spinal hypersensitivity**. A model of enhanced spinal glutamate release illustrates the possible mechanism of Ca_v_α_2_δ_1_-mediated spinal hypersensitivity. **A**. Under normal conditions, the Ca_v_α_2_δ_1 _expression level and quantum release of excitatory neurotransmitter glutamate from pre-synaptic A-fiber terminals is low so that post-synaptic deep dorsal horn neurons maintain a normal excitation threshold. As a consequence, glutamate release in response to stimuli results in a physiological activation of deep dorsal horn neurons mainly by activating AMPA/kainate receptors. The Ca_v_α_2_δ_1 _expression level at C-fiber terminals is relatively high (causing a ceiling effect for C-fiber activation), but there is little C-fiber input onto deep dorsal horn neurons at a resting state. **B**. Under a pathological condition with an elevated Ca_v_α_2_δ_1_-level at the A-fiber pre-synaptic terminals, such as post peripheral nerve injuries, the quantum release of glutamate from these terminals at a resting membrane potential is dramatically enhanced. This glutamate "leaking" causes a partial depolarization of post-synaptic deep dorsal horn neurons by activating AMPA/kainate receptors. As a consequence, these primed deep dorsal horn neurons have lowered excitation thresholds to subsequently evoked glutamate release, and also hyperexcitability due to lowered thresholds to NMDA receptor activation. Neither the partial depolarization of deep dorsal horn neurons nor the increased Ca_v_α_2_δ_1 _expression in C-fiber terminals has any detectable effect to high intensity C-fiber stimulations. **C**. Proposed potential mechanisms mediating the Ca_v_α_2_δ_1_-enhancement of spinal glutamate release.

It remains elusive that AMPA/kainate receptor antagonists only normalize behavioral hypersensitivity in the TG mice without affecting the behavioral sensitivity in the WT mice significantly. It is likely that binding of glutamate to AMPA receptors at a resting state causes a conformational change of the receptors that is linked to channel opening followed by desensitization of the receptors, a mechanism controlling glutamate-induced neuron activation under normal physiological conditions [33]. It is possible that DNQX has lower binding affinity to AMPA receptors at the conformation under physiological conditions, thus, is less effective in blocking AMPA receptors. In the transgenic mice or under a pathological condition in which pre-synaptic glutamate release is enhanced, the AMPA receptor channels could be in a different conformational state due to partial membrane depolarization and channel opening [33], a primed state as suggested in Fig. 7B. DNQX could have a higher affinity to this state, thus, be more effective in blocking AMPA receptors in the TG mice or pathological conditions. Alternatively, glutamate activation of kainate receptors at the pre-synaptic sensory neuron terminals can reduce glutamate release [34], and that at spinal interneurons can induce inhibitory transmitter release [35], which together help to terminate transient activation of post-synaptic dorsal horn neurons under normal physiological conditions. This heterosynaptic regulation is transient due to kainate receptor desensitization [35]. It is possible that this heterosynaptic regulation is less sensitive to DNQX. However, in the TG mice or under a pathological condition in which pre-synaptic glutamate release is enhanced, prolonged activation of kainate receptors at inhibitory interneurons can enhance GABA release that may activate pre-synaptic GABA_B _autoreceptors, which in turn, reduces potential-dependent inhibitory transmitter release and causes disinhibition [35]. It is possible that DNQX is more effective in blocking this prolonged activation of kainate receptors. Finally, it is possible that the WT mice are relatively insensitive to (Aβ-mediated) mechanical stimulation, possibly due to the low level Ca_v_α_2_δ_1 _expression in large neurons as discussed previously, so that it is difficult to detect any change in mechanical sensitivity. These possibilities may be not mutually exclusive.

Nevertheless, findings from this study allow us to propose the following mechanism of Ca_v_α_2_δ_1_-mediated tactile allodynia (Fig. 7): At the resting state under normal synaptic functions, the expression levels of Ca_v_α_2_δ_1 _in large DRG neurons and A-fiber terminals are relatively low, so as the rate of quanta glutamate release from A-fiber terminals that maintains a normal neuronal excitation threshold to innocuous stimulation. The Ca_v_α_2_δ_1 _expression levels in small DRG neurons and C-fiber terminals are relatively high, but may have little effect on glutamate release from C-fiber terminals at a resting state due to the lack of C-fiber inputs. This high level of basal Ca_v_α_2_δ_1 _expression in small DRG neurons may set up a ceiling effect for the influence of Ca_v_α_2_δ_1 _on WDR neuron excitability to C-fiber or windup stimulations (discussed below).

Mild pre-synaptic membrane depolarization in response to an action potential leads to the opening of VGCC, triggering calcium influx and a quantum release of excitatory neurotransmitter glutamate. Binding of glutamate to post-synaptic AMPA receptors at a resting state causes a conformational change of the receptors that is linked to channel opening, which is terminated by desensitization of the receptors [33]. In addition, glutamate activation of kainate receptors at the pre-synaptic sensory neuron terminals can reduce glutamate release [34], and that at spinal interneurons can induce inhibitory transmitter release [35]. This heterosynaptic regulation, which is transient due to kainate receptor desensitization, also plays a role in terminating transient activation of post-synaptic dorsal horn neurons under normal physiological conditions [35]. Under normal physiological conditions, released glutamate is insufficient in activating post-synaptic NMDA receptors (Fig. 7A).

After peripheral nerve injuries, injury-induced Ca_v_α_2_δ_1 _upregulation in DRG neurons, more so in large DRG neurons, leads to increased axonal transport of Ca_v_α_2_δ_1 _proteins to central pre-synaptic terminals, which in turn causes an increased rate of basal release of glutamate through a yet identified mechanism. This "glutamate leaking" causes partial sensitization of deep dorsal horn neurons by activating mainly AMPA/kainate receptors at the resting state. These "sensitized" dorsal horn neurons therefore have lowered excitation thresholds to a subsequent release of glutamate in response to innocuous or mild nociceptive stimuli that could evoke further conformational change of the AMPA/kainate receptors and prolonged channel opening, leading to spinal hyperexcitability. In addition, signals of mild noxious stimulation could be further amplified in these sensitized dorsal horn neurons due to their low thresholds to NMDA receptor activation, leading to exaggerated painful sensations (Fig. 7B). These hyperexcitability states are sensitive to antagonists of AMPA/kainate and NMDA receptors. However, neither the partial neuron sensitization by glutamate leaking from A-fiber terminals nor the increased Ca_v_α_2_δ_1 _expression in C-fiber terminals has any detectable effect on WDR neuron excitability to high intensity C-fiber stimulations (the ceiling effect discussed above).

The detail mechanisms regarding how elevated Ca_v_α_2_δ_1_mediates enhanced pre-synaptic glutamate release remain elusive (Fig. 7C). Based on in vitro data indicating that Ca_v_α_2_δ_1 _expression is required for normal membrane expression of functional VGCC [36-40], which is critical in mediating pre-synaptic neurotransmission, it is possible that elevated Ca_v_α_2_δ_1 _leads to an enhanced VGCC expression at the pre-synaptic terminals. However, there is no direct evidence so far to support this hypothesis. Alternatively, recent findings in drosophila point to the direction that Ca_vα2δ _plays a critical role in formation of pre-synaptic structures and active zones related to pre-synaptic neurotransmission [41]. Indeed, it has been proposed that the ability of descending excitatory 5HT3 mediated projections to induce a state-dependency in the actions of gabapentin may also occur with the monoamine receptor interacting with pre-synaptic mechanisms at the spinal level [16]. Thus, it is possible that increased Ca_v_α_2_δ_1 _causes enhanced synaptic strength and/or synapse numbers by promoting formation of more active zones through a yet identified mechanism. These two possibilities may not be mutually exclusive, and deserve further investigation.

## Conclusion

In conclusion, findings from this study support that elevated Ca_v_α_2_δ_1 _causes spinal hypersensitivity and tactile allodynia through at least partially enhanced pre-synaptic glutamate release, which may lead to sensitization of post-synaptic dorsal horn neurons by lowering their thresholds to innocuous stimuli. This spinal sensitization may contribute to the neuropathic pain states derived from increased pre-synaptic Ca_v_α_2_δ_1 _expression.

## Methods

### Animals

Transgenic mice over-expressing the Ca_v_α_2_δ_1 _gene were generated with a transgenic vector containing the mouse brain Ca_v_α_2_δ_1 _cDNA (Genbank accession number U73484) down-stream of a 6.5 kb murine *thy-1.2 *gene extending from the promoter region to the intron after exon 4 without exon 3 and its flanking introns as described previously [42]. Thy-1 is a member of the immunoglobulin superfamily that is expressed in both neuronal and non-neuronal tissues [43], and deletion of exon 3 and its flanking introns has been shown to eliminate expression in non-neuronal cells [44]. Only adult male TG mice and their WT littermates were used for the experiments. All the mice were fertile, appeared normal with respect to grooming, social interactions and feeding, and showed no signs of abnormality or any obvious motor defects, tremor, seizure, or ataxia [8].

All animal care and experimental procedures were performed based on protocols approved by the Institutional Animal Care Committees of the University of California, Irvine, University College London and Indiana University.

### Neuropathic lesions

The surgical procedure of SNL was performed as described [45]. Briefly, under isoflurane anesthesia, the left L5 spinal nerve of mice were exposed and ligated with a silk suture distal to DRG and proximal to their conjunction to form the sciatic nerve. Sham operations were performed in the same way except that spinal nerves were not ligated.

### Behavioral assays

Behavioral response to mechanical stimulation was tested blindly as described before and post drug treatments [8]. Briefly, mice were allowed to acclimatize for 1 hr in a clear plastic cage with a wire mesh bottom. The 50% paw withdrawal thresholds (PWT) to calibrated von Frey filament (Stoelting, Wood Dale, IL) stimulation were determined using a modified up-down method of Dixon [46]. A series of filaments, starting with one that has a buckling weight of 0.41 g, were applied in consecutive sequence to the plantar surface of the left hindpaw with a pressure causing the filament to buckle. Paw lifting within 5 s was considered a positive response and led to the use of the next weaker filament. Absence of a paw lifting after 5 s led to the use of the next filament with increasing weight. The 50% paw withdrawal threshold was calculated as described before [2] from the resulting scores of six measurements starting from the one prior to the first positive response; or until four consecutive positive (assigned a score of 0.01 g) or five consecutive negative (assigned a score of 3 g) responses had occurred. Paw withdrawal thresholds from each hindpaw of a SNL mouse were recorded separately, or averaged paw withdrawal thresholds from both hindpaws of the WT and injury-free Ca_v_α_2_δ_1 _TG mice were used for comparing sensitivities between injury and non-injury side in SNL mice or between injury-free WT and TG mice, respectively, with or without drug treatment.

### Drug administration

Drug working solutions were prepared in sterile saline before intrathecal injections between lumbar regions 5–6 (5 μL/mouse) [47]. In some cases that multiple drugs were used in the same group of animals, a three-day drug-free period was introduced between each drug to ensure that the previous drug was completely out, even though all the drug effects in this study were terminated within 24 hr post injection.

### Electrophysiological recordings in dorsal horn neurons in vivo

Recordings were performed by examiners blinded to the mouse genotype. Animals were placed in a stereotaxic frame under anesthesia and the L3–L6 spinal segments were exposed with a laminectomy. Extracellular recordings were made from individual wide dynamic range neurons using parylene coated tungsten electrodes (A-M Systems, USA). Neurons were visualized, isolated, and discriminated based on their spike amplitudes and waveforms. All neurons used for recording had receptive fields over the hindpaw. Depth of the neuron was measured from the surface of the spinal cord. Spontaneous activity was quantified over a period of at least 100 s in the absence of any evoked stimulus on isolation of a neuron.

Electrical stimulation was given via two needles inserted into the receptive field and the evoked activity was characterized and quantified as follows. A train of 16 stimuli was given (2 ms pulse duration, 0.5 Hz at three-times the C-fiber threshold), and the evoked neuronal responses were quantified using online analysis (Spike 2 software, CED Cambridge UK). The evoked neuronal responses were superimposed as a post-stimulus histogram and responses identified – based on fiber conduction velocity and the resulting latency – as C-fiber-evoked (50–250 msec) action potentials. Neuronal responses occurring after the C-fiber latency band of the neuron (250–800 ms) were classed as post discharge. The 'input' (non-potentiated response) was determined as: Input = [action potentials (50–800 ms) evoked by first pulse at 3 times C-fiber threshold] × total number of pulses.

### Electrophysiological recordings in dorsal horn of spinal cord slices

Recordings were performed by examiners blinded to the mouse genotype. The animals were anesthetized with ketamine-HCl (1 mg/kg, i.p.) and the spinal cord was extracted by hydraulic extrusion. Transverse slices (330 μm) were cut from the L5 lumbar enlargement using a vibratome (VT1000S; Leica, Nussloch, Germany) in an ice-cold (4°C) sucrose solution containing (in mM): 230 sucrose, 26 NaHCO_3_, 2.5 KCl, 1.25 NaH_2_PO_4_, 0.5 CaCl_2_, 10 MgSO_4_, 10 glucose, pH 7.4, 290–305 mOsm/L, equilibrated with 95% O_2 _and 5% CO_2_. The slices were maintained in an artificial cerebrospinal fluid (ACSF) containing (in mM): 130 NaCl, 3 KCl, 2 CaCl_2_, 2 MgCl_2_, 1.25 NaH_2_PO_4_, 26 NaHCO_3_, and 10 glucose, pH 7.4, 295–305 mOsm/L. The ACSF was continuously equilibrated with 95% O_2 _and 5% CO_2_, and slices were incubated for > 1 h before recording. Recording electrodes were prepared from borosilicate glass (World Precision Instruments, Sarasota, FL) using a horizontal electrode puller (P-97; Sutter Instruments, Novato, CA). Electrodes had resistances of 2–4 MΩ when filled with an intracellular solution containing (in mM): 92 CsMeSO_4_, 43 CsCl, 1 MgCl_2_, 2 EGTA, 5 TEA, 10 HEPES and 2 Mg-ATP, pH 7.4, 295–300 mOsm/L. Oxygenated ACSF was used as bath solution, and the flow rate was adjusted to 2–3 mL/min. Depth of the neuron was measured from the surface of the spinal cord and neurons in the deep dorsal horn were visualized with an infrared-differential interference contrast (DIC) microscope (BX50WI; Olympus Optical, Tokyo, Japan) and a CCD camera. All recordings were performed at 25 ± 1°C with an Axopatch 200B amplifier (Molecular Devices, Foster City, CA). During whole-cell recordings, series resistance (8–15 MΩ) was monitored periodically, and cells with > 15% changes were excluded from the analysis. Signals were filtered at 1 kHz and digitized at a sampling rate of 5 kHz using a data-acquisition program (Axograph 4.6; Molecular Devices).

To record mEPSCs, tetrodotoxin (1 μM) and bicuculline (30 μM) were continuously applied in the bath solution. At a holding potential of -70 mV, the recorded mEPSCs were mainly mediated by AMPA/kainate receptors because application of AMPA/kainate receptor antagonist CNQX (10 μM) could reduce mEPSCs by 91.3 ± 5.3% and 93.9 ± 3.3% in WT and TG neurons (n = 5 in each group), respectively. In the presence of CNQX (10 μM), NMDA receptor-mediated mEPSCs were recorded at a holding potential of +50 mV.

### Statistical Analyses

Indicated significant changes were defined by the two-tailed *p *values as determined with unpaired Student's *t *test, or two-way ANOVA followed by Bonferroni post-tests.

## Competing interests

The authors declare that they have no competing interests.

## Authors' contributions

DN participated in the design, carried out the behavioral pharmacology studies, and participated in drafting the manuscript. PD participated in the design and carried out the electrophysiology studies in spinal cord slices. EAM participated in the design and carried out the electrophysiology studies in dorsal horn neurons in vivo. DSK participated in the experimental design and animal model studies. GF participated in the design and generation of the transgenic mice. AHD participated in the experimental design, coordination and data analysis of the in vivo electrophysiology studies, and in drafting the manuscript. ZCX participated in the experimental design, coordination and data analysis of the electrophysiology studies in spinal cord slices, and in drafting the manuscript. ZDL conceived of the study and participated in its design, coordination and drafted the manuscript. All authors read and approved the final manuscript.
